# Musculoskeletal effects of obesity and bariatric surgery – a narrative review

**DOI:** 10.20945/2359-3997000000551

**Published:** 2022-11-10

**Authors:** Narriane Holanda, Nara Crispim, Ingrid Carlos, Taíssa Moura, Eduardo Nóbrega, Francisco Bandeira

**Affiliations:** 1 Universidade Federal da Paraíba Hospital Universitário Lauro Wanderley Departamento de Endocrinologia e Metabologia João Pessoa PB Brasil Departamento de Endocrinologia e Metabologia, Hospital Universitário Lauro Wanderley, Universidade Federal da Paraíba, João Pessoa, PB, Brasil; 2 Universidade de Pernambuco Hospital Agamenon Magalhães Faculdade de Ciências Médicas Recife PE Brasil Divisão de Endocrinologia e Diabetes, Hospital Agamenon Magalhães, Faculdade de Ciências Médicas, Universidade de Pernambuco, Recife, PE, Brasil

**Keywords:** Obesity, bariatric surgery, bone, sarcopenia

## Abstract

Obesity affects several areas of the human body, leading to increased morbidity and mortality and the likelihood of other diseases, such as type 2 diabetes mellitus, cardiovascular diseases and musculoskeletal disorders. These conditions predispose to bone fractures and sarcopenic obesity, defined by the presence of an obesity-associated decrease in muscle mass and strength. Both bone fragility and sarcopenic obesity disease are consequences of several factors, such as a low degree of chronic inflammation, insulin resistance, hormonal changes, nutritional deficiencies, ectopic fat deposits and sedentary lifestyle. The diagnosis of obesity-related musculoskeletal disorders is limited by the lack of sarcopenia criteria and lower accuracy of bone mineral density measurement by dual-energy X-ray absorptiometry in overweight people. Reducing body weight provides undeniable benefits to this population, however treating cases of severe obesity with bariatric surgery can cause even greater damage to bone and muscle health, especially in the long term. The mechanisms involved in this process are not yet fully understood, but factors related to nutrient malabsorption and mechanical discharge as well as changes in gut hormones, adipokines and bone marrow adiposity should be taken into account. Depending on the surgical technique performed, greater musculoskeletal damage may occur, especially in cases of malabsorptive surgeries such as Roux-en-Y gastric bypass, when compared to restrictive techniques such as sleeve gastrectomy. This difference is probably due to greater weight loss, nutrient malabsorption and important hormonal changes that occur as a consequence of the diversion of intestinal transit and loss of greater absorptive surface. Thus, people undergoing bariatric procedures, especially malabsorptive ones, should have their musculoskeletal health supervised to allow early diagnosis and appropriate therapeutic interventions to prevent osteoporotic fractures and preserve the functionality of the skeletal muscles. Arch Endocrinol Metab. 2022;66(5):621-32

## INTRODUCTION

Obesity is a chronic disease characterized by excessive accumulation of fat, generating an inflammatory state that culminates in increased morbidity (type 2 diabetes mellitus, cardiovascular diseases, kidney disease and musculoskeletal disorders) and mortality (1,2). Its prevalence has increased in recent years, with a forecast of one billion people worldwide living with obesity by 2030 (3).

Bariatric surgery (BS) is the most effective treatment for severe obesity and has become commonly performed around the world with undeniable benefits, both in reducing body weight and improving metabolic and cardiovascular conditions (4–6). However, such a procedure can cause damage to bone and muscle health, especially in the long term (5,6).

Vertical gastrectomy or sleeve (VG) and Roux-en-Y gastric bypass (RYGB) comprise more than 80% of bariatric procedures performed worldwide (7). Both techniques seem to increase the bone and skeletal muscle mass (SMM) loss over time, but malabsorptive or mixed surgeries result in greater musculoskeletal damage, probably due to greater weight loss, nutrient malabsorption and important hormonal changes when compared to restrictive surgeries (8,9). However, data that compare the two techniques and their real impact on musculoskeletal health directly are still scarce.

Several pathophysiological mechanisms are involved in the skeletal and muscle changes observed after BS. In this article, we will present a narrative review on the main aspects of the pathophysiology of musculoskeletal changes related to obesity per se and BS, in addition to bone and muscle health care after these procedures. A narrative review was carried out using the electronic literature available in the databases LILACS, CENTRAL, Web of Science, Embase and PubMed/MEDLINE. Keywords were descriptors for obesity, bariatric surgery, bone and sarcopenia, including articles from the 2000s to the present.

## BONE AND MUSCLE CHANGES RELATED TO OBESITY

The relationship between bone, fat and muscle tissue begins with the genesis of adipocytes, osteoblasts and myocytes, which derive from the same mesenchymal precursor cell (MPC). Faced with a hostile environment, such as a low degree inflammation caused by obesity, MPC seems to favor the differentiation to the adipogenic lineage instead of the others. This trans-differentiation to bone and muscle lineage is influenced by local, systemic and environmental factors. In addition to obesity, aging and sarcopenia (SARC) favor adipogenesis and suppression of osteoblastogenesis and myogenesis (10).

Furthermore, depending on where the fat tissue is stored, it can have different effects on bones and muscles. While subcutaneous fat results in anabolic stimulus secondary to mechanical overload, production of leptin, adiponectin and peripheral estrogenic aromatization, visceral fat has a pro-inflammatory action that increases bone resorption and myocyte degeneration. Intramuscular fat, in addition to reducing the beneficial effect of mechanical overload, promotes inflammation, muscle dysfunction with the consequent increased risk of falls (11).

Obesity is also associated with several nutritional deficiencies. The high prevalence of vitamin D insufficiency in this population results from the sequestration of vitamin D in visceral fat, low exposure to sunlight, low intake of foods containing vitamin D and decreased hepatic synthesis of substrates for the formation of 25-hydroxyvitamin D (25OHD). Furthermore, an independent relationship exists between obesity and increased parathyroid hormone (PTH), which is exacerbated by the reduction of vitamin D, contributing to the presence of secondary hyperparathyroidism (SHPT) well before BS (11).

## RISK OF FRACTURES AND OBESITY

For a long time, obesity was believed to be protective for bone, mainly due to the fact that people living with obesity have higher bone mineral density (BMD), whereas people who are underweight are classically at greater risk of fractures (12). This observation was further supported by the anabolic effect of mechanical loading of body weight on bone tissue and by the well-known positive action of estrogen on bone (13). However, despite the increase in BMD, studies have shown that body mass index (BMI) is positively correlated with the risk of fractures in this population, especially in peripheral sites such as the proximal humerus, thigh and ankle (14,15).

Besides an increased inflammatory state due to obesity, some genetic factors associated with weight gain may predispose to osteoporosis; beyond acceleration of osteoblast agingand alterations in the intestinal microbiota (11,16) that may contribute to bone fragility. From a hormonal point of view, obesity is associated with endocrine disruption, especially involving adipokines, leptin, adiponectin, sclerostin and irisin, which play an important role in musculoskeletal metabolism (17). In addition, serum 25OHD levels are reduced in obese individuals compared to non-obese individuals, which may contribute to negative osteometabolic outcomes alone or by stimulating the elevation of PTH levels (18). Together, these mechanisms promote increased bone turnover and reduced BMD and SHPT, which predisposes this population to bone fractures.

Finally, the higher fracture risk in obese individuals can also be explained by the higher frequency of falls due to the imbalance that excess weight promotes, causing them to fall sideways or backwards, while the increase in fat in the hips and abdomen can protect them against fractures in the axial skeleton (16).

## BONE ASSESSMENT IN OBESITY

Although dual-energy X-ray absorptiometry (DXA) is considered the standard test for quantification of bone mass, we must be careful when interpreting its results in people with obesity.

Areal BMD measured by DXA is higher in people with obesity, but studies indicate that the higher BMI and soft tissue thickness can cause overestimated results with error rates of up to 20%, due to the overlapping of abdominal fat (4,19).

It is worth remembering that the concept of bone fragility involves, in addition to quantity, the quality of the bone, which is more difficult to measure. These measurement errors can be minimized using the quantitative computed tomography (QCT), which has shown greater accuracy in measuring volumetric bone mass, or the evaluation of bone microarchitecture through high-resolution peripheral quantitative computed tomography (HR-pQCT) (20). On the other hand, the trabecular bone score (TBS) has a decreased accuracy in patients with obesity. It has been used to assess bone microarchitecture, but both BMI and the excess of subcutaneous fat may limit its precision (21).

## SARCOPENIC OBESITY

SARC is a condition characterized by a progressive and generalized musculoskeletal disorder diagnosed by low muscle strength associated with low muscle mass or quality (22). Elderly people are more susceptible to SARC due to the decrease in SMM and muscle function with advancing age (22,23). However, this loss of SMM does not depend only on age (24,25).

Several risk factors can accelerate the onset of SARC in obese individuals, such as oxidative stress, inflammation and insulin resistance, particularly in the presence of metabolic complications and other comorbidities. In addition, other factors, such as acute and chronic diseases as well as cycles of weight loss and gain, may contribute to the loss of SMM (26).

Therefore, sarcopenic obesity (SO), a condition characterized by the association of obesity with SARC, has received considerable attention in recent years because it is related to several negative clinical outcomes. In the elderly, SO increases the risk of disability (27), falls, osteoporosis, fractures (22,23), metabolic changes (26), arterial stiffness (28,29), non-alcoholic fatty liver disease (30), complications from cancer (31), worse cognitive performance (32) and increased mortality (29,33,34). Among obese adults, it can lead to insulin resistance (35), metabolic syndrome (35), diabetes mellitus (36,37), systemic arterial hypertension (37), difficulties in activities of daily living (38) and increase mortality (33).

Recently, another condition has gained interest from researchers and clinicians. Osteosarcopenic obesity is characterized by the association of osteopenia or osteoporosis with SARC and obesity. In addition to the complications listed above, this can increase the risk of frailty and predisposes one to lower physical performance (39,40).

The heterogeneity and lack of consensus in the diagnostic criteria for SO impact its prevalence directly (41,42). SO is more prevalent in the elderly, and its diagnostic criteria may differ according to gender. The definition of SO using SMM or appendicular SMM (ASMM) adjusted for height tends to underestimate its prevalence, especially in women (43–45). When muscle mass is adjusted for weight, the prevalence tends to be higher (46), especially in females, regardless of the definition of obesity used (body fat percentage, BMI or waist circumference) and age (adults or elderly people) (25,26,35,47). When the definition of SO involves muscle function, the prevalence of SARC tends to be lower (26).

SO screening is recommended in individuals with obesity according to BMI or increased WC (according to ethnicity) associated with surrogate parameters for SARC (clinical symptoms, clinical suspicion, age > 70 years, chronic diseases, acute diseases/nutritional events or the SARC-F questionnaire for the elderly). The diagnosis must be confirmed by the presence of low muscle strength associated with low muscle mass and high body fat percentage (BFP). When muscle mass is estimated by DXA, the ASMM adjusted for weight must be used, and when it is estimated by bioimpedance (BIA), the SMM adjusted for weight must be used (48).

Some aspects of the classic characterization of SARC in the elderly might not be appropriate for individuals with obesity. First, gait speed would not be so accurate, since, due to joint involvement, the individual may not be able to perform that test. Second, young individuals with obesity may have normal muscle strength, but a diagnosis of low muscle mass would bring negative clinical outcomes (48). It has been suggested to assess the severity of SO in two stages. In stage 1, there are no complications associated with this condition; in stage 2, complications such as metabolic diseases, disability, cardiovascular and respiratory diseases are present (48).

## MUSCLE ASSESSMENT IN OBESITY

### Muscle mass

Magnetic resonance imaging (MRI) accurately quantifies the SMM, in addition to assessing the infiltration of fat into the muscle (distinguishes intra- and extracellular fat). Despite its high reliability, due to costs, it has been used more often in clinical research (49).

Computed tomography (CT) is less sensitive than MRI. It is capable of evaluating intermuscular fat, but due to radiation issues, it is used in clinical practice as a second option, such as when investigating other conditions (49).

In view of the limited availability of MRI and CT, it has recently been recommended that SMM in obese individuals be assessed by DXA (or BIA, as a second option). Both methods have limited applications to obesity, including the lack of direct measurement of the SMM. In DXA, the assessment of lean mass, which includes non-muscle tissue, leads to discrepancies between body composition and functional parameters. In BIA, the use of specific equations for calculations requires validation and cutoff points, which can differ significantly between studies. In addition, BMI > 34 kg/m^2^ can lead to underestimation of fat mass and overestimation of fat-free mass (48).

### Muscle function

Muscle function is represented by strength and physical performance. Strength should be assessed by handgrip strength (HPF) or a chair-rising test. The assessment of physical performance has its limitations in obese individuals due to joint impairment (48).

## BONE CHANGES AFTER BARIATRIC SURGERY

### Bone mass, microarchitecture and bone remodeling markers

Progressive reduction in BMD and increase in bone turnover markers (N-terminal procollagen type 1 pro-peptide [P1NP] and type I collagen C-telopeptide [CTX]) occur after BS. This increase occurs early and dramatically, may remain for several years after the procedure (8,50) and coincides with bone loss at the appendicular and axial skeleton, especially after RYGB (8,51).

A recent randomized clinical trial found a greater reduction in BMD in the femoral neck, total hip and lumbar spine, in addition to a greater increase in bone remodeling markers in the group that underwent RYGB compared to VG, despite stabilization of weight loss (52). Corroborating these findings, our group also found a greater reduction in femoral neck and total body BMD in patients undergoing RYGB compared to VG, which were associated with an increase in serum CTX and alkaline phosphatase levels (8).

### Secondary hyperparathyroidism and vitamin D levels

SHPT is more frequent in the obese population after BS than in the general population, especially after RYGB (8,53). After BS, despite adequate calcium, vitamin D supplementation and weight loss, serum calcium and 25OHD levels are often low or at the lower limit of normal, while PTH levels are independently elevated (8,53–55). Furthermore, in most studies, serum calcium levels remain normal throughout the follow-up period, and this occurs at the expense of high bone turnover. So, serum calcium measurements may not be a good marker of postoperative calcium deficiency (8,54).

### Risk of bone fractures after bariatric surgery

Most current evidence suggests that BS increases the risk of fractures (56–61), particularly after malabsorptive or mixed procedures, when compared to restrictive procedures (62–64).

Two recent meta-analyses have evaluated the risk of fractures according to BS procedure. One of them, from our group, showed an overall risk of fractures 1.2 times higher in patients undergoing BS compared to obese patients undergoing conservative treatment, this risk being higher in patients undergoing RYGB compared to VG [RR 1 .77 (95% CI 1.48-2.12, p < 0.00001)] (65). Saad and cols. also demonstrated that the risk of fracture associated with malabsorptive procedures was higher when compared to patients undergoing restrictive surgery [RR 1.61 (95% CI 1.42-1.83, p < 0.00001)] (66). Table 1 provides a summary of the main observational and randomized studies that evaluated the risk of fracture associated with BS.

**Table 1:** Fracture risk after bariatric surgery

Author, Origin, Year	Type of study	Inclusion criteria	Types of bariatric surgeries performed in the intervention groups	Risk ratio (95% CI) – Fracture
Lalmohamed, UK, 2012 (98)	Retrospective cohort	BMI ≥ 30 kg/m^2^ with bariatric surgery record	Adjustable gastric banding Gastric bypass Others	0.89 (0.60-1.33)
Nakamura, USA, 2014 (59)	Retrospective cohort	Patients undergoing bariatric surgery. Pathological fractures were excluded	Gastric bypass Others	2.3 (1.8-2.8) – Any site
Douglas, UK, 2015 (99)	Retrospective cohort	Patients registered in the database with bariatric surgery and obese patients matched without surgery	Gastric band Gastric bypass VG Others	1.26 (0.79-2.01)
Lu, Taiwan, 2015 (60)	Retrospective cohort	Prevalent morbid obesity, excluding patients with a previous diagnosis of fracture or osteoporosis	Malabsorptive procedures Restrictive procedures	1.21 (1.01-1.44) –Any fracture 1.47 (1.01-2.15) –Malabsorptive procedures x control 1.17 (0.97-1.41) –Restrictive procedures x control
Rousseau, Canada, 2016 (61)	Retrospective cohort	Severe obesity undergoing bariatric surgery	Adjustable gastric banding VG Gastric bypass Biliopancreatic diversion	1.44 (1.29-1.59) – Bariatric group x Non obese control 1.38 (1.23-1.55) – Bariatric group vs. obese control
Axelsson, Sweden, 2018 (58)	Retrospective cohort	BMI ≥ 30 kg/m^2^ undergoing bariatric surgery divided into groups with and without diabetes and compared to obese controls	Gastric bypass with and without diabetes	1.26 (1.05-1.53) – with diabetes 1.32 (1.18-1.47) – without diabetes
Fashandi, USA, 2018 (56)	Retrospective cohort	Patients undergoing bariatric surgery and corresponding cohort of non-surgical obesity	RYGB Gastric banding VG Others	2.36 (1.72-2.23) – Bariatric surgery x control 2.17 (1.04-4.52) – RYGB x VG
Javanainen, Finland, 2018 (57)	Retrospective cohort	BMI ≥ 35 kg/m^2^, ages 18 to 65 years, and previous failed weight loss attempts through non-surgical obesity programs (bariatric surgery patients)	RYGB VG	5.49 (1.76-17.15) – Bariatric surgery x control
Ahlin, Sweden, 2020 (64)	Non randomized interventional study	Age 37-60 years and BMI > 34 kg/m^2^ for men and > 38 kg/m^2^ for women	Adjustable or nonadjustable gastric banding Vertical banded gastroplasty Gastric bypass	2.58 (2.02-3.31) – Gastric bypass x control 2.15 (1.66-2.79) - Gastric bypass x Vertical banded 1.20 (1.00-1.43) – Vertical banded gastroplasty x control
Khalid, EUA, 2020 (63)	Retrospective cohort	Patients classified as eligible for bariatric surgery who did not undergo bariatric surgery or underwent RYGB or VG	RYGB VG	1.79 (1.55-2.06) – RYGB x VG 0.95 (0.84-1.07) – RYGB x control 0.53 (0.46-0.62) – VG x control
Paccou, France, 2020 (62)	Retrospective cohort	Patients undergoing bariatric surgery, aged between 40 and 65 years, BMI ≥ 40 kg/m^2^, were matched with controls	VG Gastric bypass Vertical banded gastroplasty Gastric banding	1.22 (1.08-1.39) – Bariatric surgery x control 1.70 (1.46-1.98) – Gastric bypass x control 0.95 (0.79-1.14) – VG x control
Zhang 2020 (100)	Meta-analysis	Studies with obese patients (BMI ≥ 30 kg/m^2^) undergoing bariatric surgery compared to a control group, with assessment of bone fracture outcome	Malabsorptive procedures Restrictive procedures	1.41 (1.22-1.63)
Chaves 2021 (65)	Meta-analysis	Studies with patients ≥ 18 years, BMI ≥ 30 kg/m^2^ and minimum follow-up of one year, compared to a non-surgical control group, matched by at least sex and age	Malabsorptive procedures Restrictive procedures	1.20 (1.15-1.26)
Chin, Taiwan, 2021 (97)	Retrospective cohort	Obese patients, aged between 18 and 55 years, divided into two groups	Malabsorptive procedures Restrictive procedures	1.693 (1.077-2.661) – Bariatric surgery x general population 0.774 (0.539-1.110) – Bariatric surgery x non-surgical group
Saad 2022 (66)	Meta-analysis	Studies in adults (> 18 years) with obesity (BMI ≥ 30 kg/m^2^), undergoing different types of bariatric surgery	Malabsorptive procedures Restrictive procedures	1.61 (1.42-1.83)

CI: confidence interval; BMI: body mass index; RYGB: Roux-en-Y gastric bypass; VG: vertical gastrectomy or sleeve; DM: diabetes mellitus.

Surgical treatment for obesity also seems to change the fracture pattern, moving from peripheral sites (lower and upper limbs) to classic osteoporotic sites, such as the spine and femur (61,65).

### Pathophysiology of musculoskeletal changes after bariatric surgery

The progressive reduction in BMD observed after BS may be influenced by the reduction in the mechanical load imposed on the bone, especially in the first postoperative year, a period of more intense weight loss (8,67). De Holanda and cols. (2021) demonstrated a significant association between weight loss and bone mass decline at all sites (8). Despite this, as the loss of BMD persists after the stabilization of weight loss, the idea that other changes in addition to the reduction of mechanical overload contribute negatively to bone metabolism is reinforced (68).

Several micronutrients and macronutrients are important for the maintenance of bone health and, for the most part, are absorbed in the jejunum and ileum. With the exclusion of this part of the intestine in some surgical techniques, associated with reduced food intake, nutritional deficiencies become quite common, especially after malabsorptive procedures (54,69) in which the increase in CTX is associated with lower absorption of calcium. These observations reinforce the importance of nutritional factors in the pathophysiology of post-bariatric bone disease (54).

Deficiencies of other micronutrients, such as magnesium, also play an important role in bone metabolism. Reduced magnesium levels are associated with reduced BMD through interference with PTH secretion and the action of this hormone on bone. A previous study described a 32% prevalence of hypomagnesemia in patients who underwent RYGB (70).

Finally, protein intake is often inadequate due to the reduced caloric requirement in their diets and the fact that obese patients are often intolerant of this nutrient (11).

Changes in the anatomy of the gastrointestinal tract and weight loss caused by BS lead to complex hormonal changes that alter the balance between bone formation and resorption. Ghrelin, GLP-1, GIP, leptin, insulin, estrogen and testosterone are associated with positive effects on bone formation, while peptide YY and adiponectin are negatively correlated with bone health (71).

The reduction in adipose tissue after the surgical treatment of obesity causes a reduction in leptin levels and an increase in adiponectin levels, and these changes may lead to an increase in bone resorption (72,73). In addition, increased levels of peptide YY are associated with increased markers of bone turnover and a reduction in BMD, and reductions in ghrelin and insulin can negatively affect bone remodeling (74). Regarding GLP-1, GIP, estrogen and testosterone, data are still lacking on their effects on bone health after BS.

Sarcopenia is one of the complications of bariatric procedures that can negatively influence bone health, since the maintenance of bone mass and architecture undergoes changes in the face of reduced appendicular skeletal mass (75,76). This fact may be related to the increase in sclerostin levels, which has been associated with weight loss after BS due to the influence of mechanical loading on the levels of this protein, which inhibits bone formation via the Wnt/*β*-catenin pathway (77).

### Muscle changes after bariatric surgery

As with bone mass, BS can lead to a significant reduction in skeletal muscle mass, especially in the first two years after the procedure (78). Furthermore, the cycle of weight loss and weight regain, common in these individuals, is associated with the return of fat mass, often without recovery of lean mass (79). Thus, most of these patients still maintain high PGC, despite significant weight loss after this surgery (80).

Body composition changes are characterized by a marked decrease in body fat, especially in the first year of follow-up, but also by a significant reduction in SMM, usually up to the second year postoperatively (80). These factors are aggravated by the lack of physical-resistance exercise and inadequate caloric/protein intake (11). Although weight loss can last for more than five years after BS, muscle loss occurs mainly during the first year postoperatively (81–83). For this reason, a multidisciplinary support is essential in the perioperative period, since muscle mass affects the basal metabolic rate, with a decrease of 1.95 kcal per kilo of lean mass lost (83).

Despite the evidence regarding lean mass loss after BS (81–84), there is a lack of data about which subgroups of individuals are at greater risk. The magnitude of insulin resistance and baseline fat-free mass (FFM) may be predictors for this outcome (85).

Regarding the differences in body composition according to the type of BS, a recent prospective study with 2 years of follow-up, involving 85 patients undergoing RYGB and VG, concluded that the loss of FFM was 21 ± 14% of total weight loss (TWL) and occurred regardless of gender, age or surgical technique, despite the higher percentage TWL in the RYGB group *versus* VG. There were no differences between groups regarding body composition or biochemical profile (85).

### Bariatric surgery and sarcopenia

Studies evaluating the impact of BS on muscle function are conflicting, some showing no change in muscle strength after BS (86). A review of observational studies suggests that physical performance improves after BS (87).

It is unclear whether individuals with OS before BS would be at risk for muscle dysfunction, compared to obese individuals without SARC. The non-identification of these participants regarding the presence of SARC before BS in the main studies limits their conclusions regarding the risks of muscle dysfunction after BS.

Studies evaluating SARC after BS have involved only low muscle-mass criteria (84,88,89). Measurement of the cross-sectional area of skeletal muscle in the third lumbar vertebra (SMA, cm^2^) by CT, and the skeletal mass index (SMI; SMA/m^2^) was done in one study (89). SARC was defined as SMI < 38.5 cm^2^/m^2^ for women and < 52.4 cm^2^/m^2^ for men, and 8% (n = 15) of the individuals already had SARC before BS. After one year of follow-up, 32% (n = 59), with a multivariate adjustment for male gender, SMA and SMI before surgery, were significantly correlated with the occurrence of SARC one year after surgery (89).

Notably, obesity-associated SARC increases the risk of clinical complications. A retrospective study comparing patients undergoing RYGB and VG demonstrated that sarcopenic obese subjects achieved the same weight loss and resolution of comorbidities as non-sarcopenic obese patients at 3, 6 and 12 months after BS. As limitations of this study, men and women were placed in the same group, and SARC was defined by SMM/m^2^ in the lowest tertile, which could underestimate SARC in obese patients (90).

Table 2 provides a summary of the main studies involving sarcopenic obesity in obese adults by body mass index.

**Table 2:** Studies involving sarcopenic obesity in obese adults by body mass index

Studies	Population	Obesity	Sarcopenia	Outcome	Methods	Prevalence of sarcopenic obesity
Crispim Carvalho 2019 (46)	Brazil (W) Age:(24-57 years)	BMI 42.6 kg/m^2^ *BS indication	ASMM/Weight ASMM/BMI Lowest quintile (Muscle function was assessed but not entered into the diagnosis)	Body fat percentage, handgrip strength, six-minute walk test, metabolic perfil, bone mineral density	BIA	ASMM/Weight 30.5% ASMM/BMI 20.33%
Johnson Stoklossa, Ghosh, 2017 (38)	Canadians (W/M) Age: (46.9 years)	BMI: 43.5 kg/m^2^ *BS indication	ASMM/Weight *Lowest quintile + < 2SD of the group mean	ADL difficulties	DXA	W: 22.3% M: 41.2%
Johnson Stoklossa, Sharma, 2017 (96)	Canadians (W/M) Age: (18-69 years) Mean: 46.9 years	BMI:43.5 kg/m^2^ *BS indication	– ASMM/Weight – ASMM/BMI – ASMM/fat mass (residual) – ASMM/height # – ASMM # – ASMM # Lowest quintile + < 2SD of the group mean	_	DXA	– MMEA/Weight – Newman (residual) M: 17.6%/ W:19.4% – IMM by Prado: 13.3% – MMEA adjusted for weight, BMI and fat mass: W: 12.6-84.5% M: 17.6-100% #did not identify sarcopenia
Kreidieh, 2018 (37)	Lebanon (W) Age: (33.26 years)	BMI 31.42 kg/m^2^ *Overweight and obesity	ASMM/BMI Cutoff (FINH-0.512 m^2^)	Association with DM and SAH	BIA	W: 20.1%
Mastino, 2016 (90)	Italians (M/H) Age: – Sarcopenic: 44 years – Non-sarcopenic: 47 years	BMI Sarcopenic: 41.1 kg/m^2^ Non-sarcopenic: 42.9 kg/m^2^	ASMM/height 2 *Lower tercile (Muscle function was assessed but not entered into the diagnosis)	Weight loss after BS and resolution of comorbidities (no difference)	BIA	Not reported
Poggiogalle, 2016 (35)	Italians 18-65 years Mean: 45.72 years	BMI: 37.74 kg/m^2^	ASMM/Weight x 100 ASMM/height^2^ -2 SD below young adult (20-39)	Metabolic syndrome	DXA	ASMM/Weight x 100 M: 34.8%/W: 50.1% ASMM/height^2^: M: 1%/W: 0.6%
Prado, 2014 (45)	Americans (NHANES) Age: ≥ 18 years Mean: M: 44.57 years W: 46.8 years	Fat mass/height^2^ (*Decile 50-100) *Obese subgroup by BMI	ASMM/height2 *Decile 0-49.9	_	DXA	M: 2.3% W: 0.3%
Srikanthan, 2010 (36)	Americans (NHANES) Age:< 60 years (Mean 37 years)	BMI > 30 kg/m^2^	SMM/Weight x 100	IR (HOMA-IR) HbA1c Pre-DM DM *risk for all outcomes	BIA	3.39%

NHANES: National Health and Nutrition Examination Survey; BMI: body mass index; SMM: skeletal muscle mass; IR: insulin resistance; HOMA-IR: homeostasis model assessment-insulin resistance; HbA1c: glycated hemoglobin; DM: diabetes mellitus; BIA: bioimpedance; W: women; M: man, ASMM: appendicular skeletal muscle mass; DXA: dual-energy X-ray absorptiometry; BS: bariatric surgery; ADL: activities of daily living; FNIH: Foundation for the National Institute of Health; SAH: systemic arterial hypertension.

* With the exception of the highest cut-off point for the height.

## MANAGEMENT OF OSTEOMUSCULAR HEALTH AFTER BARIATRIC SURGERY

Patients undergoing BS should have their bone density measured at spine and hip, preferably before and 2 years after the procedure. In addition, annual laboratory tests should include: serum albumin (screening for protein malnutrition) and total calcium and 25-hydroxyvitamin D (for all surgical techniques). Serum PTH, phosphorus and 24-hour urine calcium (for mixed or malabsorptive surgeries) (91,92). The use of FRAX^®^, vertebral fracture assessment (VFA) by DXA and TBS still have limited utility in this population (91). Bone resorption markers can also be used to monitor bone remodeling after BS, especially in peri- and post-menopausal women (91,93). Additional risk factors such as smoking, alcohol and long-term use of proton pump inhibitors should also be considered (91).

Oral calcium citrate supplementation is indicated, according to the type of technique used (1,200-1,500 mg/day of elemental calcium for VG, RYGB and laparoscopic adjustable gastric banding; 1,800-2,400 mg/day for biliopancreatic diversion with duodenal switch). Vitamin D should be supplemented (preferably with cholecalciferol) at a dose of 3,000 to 6,000 IU/day, aiming to maintain serum 25OHD levels between 30 and 60 ng/mL (92,94). In addition, protein intake should be adjusted, adding whey protein if necessary, reaching at least 60 g/day and up to 1.5 g/kg of ideal body weight per day, with higher targets according to individual need (92).

Regular physical exercise should also be encouraged, and moderate aerobic physical activity should be indicated (at least 150 minutes/week) associated with strength training (muscle strength and/or resistance training 2-3 times/week) (95).

If osteoporosis is diagnosed, antiresorptive agents are the first-line therapy, but before starting, appropriate therapy for calcium and vitamin D insufficiency should be given. Parenteral presentations are the first choice for treatment. The oral route may rarely be considered if there are no concerns about oral absorption of the medication or the presence of anastomotic ulcers. Denosumab may be considered if there is no response to bisphosphonate therapy or if it is poorly tolerated, but attention should be paid to the possible risk of hypocalcemia in this population (92). The use of anabolic agents such as teriparatide is limited, as they should be used only in patients who do not have SHPT (75).
